# Gonadal and Cortisol Hormone Profile among Male Chronic Khat, Marijuana, and Heroin Abuses

**DOI:** 10.1155/2019/4178241

**Published:** 2019-11-03

**Authors:** Meseret Yibrah, Abebe Edao Negesso, Atsbeha Gebregziabher, Feyissa Challa, Kissi Mudi, Feven Tesfay, Mehari Gebretsadkan, Samuel Kinde, Daniel Asmelash

**Affiliations:** ^1^Department of Medical Laboratory Science, College of Health Sciences, Mekelle University, Mekelle, Ethiopia; ^2^Department of Medical Laboratory Science, College of Health Science, Addis Ababa University, Addis Ababa, Ethiopia; ^3^HIV and TB Research Directorate, Ethiopian Public Health Institute (EPHI), Addis Ababa, Ethiopia; ^4^Department of Clinical Chemistry, College of Medicine and Health Science, University of Gondar, Gondar, Ethiopia

## Abstract

**Background:**

Drugs of abuse could interfere with the hypothalamic-pituitary-gonadal axis, causing impaired functions of the gland and associated functions of target organs. Drugs of abuse tend to cause changes in the endocrine system, and these changes could be physiological, molecular, biochemical, genetic, and cellular.

**Method:**

A case-control study design was conducted from a total of 171 male consented study participants (148 drug abusers and 23 controls). The serum gonadal and cortisol hormone levels were assayed using the electrochemiluminescence immunoassay method. Socio-demographic variables were collected using a semi-structured questionnaire by the interview method. Nonparametric statistical tools (Mann–Whitney test and median) were used to compare the groups. In all cases, *P* < 0.05 was considered statistically significant.

**Result:**

The median age for drug abusers and control groups was 27, and the age difference between drug abusers and control group was not statistically important. The median estradiol levels among chronic khat chewers (39.4 pg/ml), marijuana (44 pg/mL), and users of heroin (40.2 pg/mL) were significantly higher than control groups (23 pg/mL), *P* < 0.003. However, the median luteinizing hormone levels among chronic khat chewers (5 IU/L), marijuana users (5 IU/L), and heroin users (5.6 IU/L) were significantly lower than those of control groups (6.2 IU/L), *P* < 0.02. The median testosterone levels among chronic khat chewers (6.1 ng/mL), marijuana users (6.3 ng/mL), and heroin users (6.6 ng/mL) were significantly lower than control groups (8.0 ng/mL), *P* < 0.003. However, cortisol and follicle stimulating hormone did not show statistically significant difference between users of khat, marijuana, and heroin compared with the control group.

**Conclusion:**

The drug abuser group had significantly lower testosterone and luteinizing hormone levels compared with control groups. Drug abuse has been shown to affect gonadal hormones in an unusual physiological phenomenon. These findings reveal the need for intervention programs to solve these problems.

## 1. Background

Drugs of abuse tend to cause changes in the endocrine system, and these changes could be physiological, molecular, biochemical, genetic, and cellular [1]. Addiction often interferes with the hypothalamic-pituitary-endocrine (HPG) axis, causing the secretion to change in the endocrine glands [2]. Hypogonadism in men results from failure of the testes to produce adequate levels of testosterone and a normal number of sperms due to a disruption of the HPG axis. The pathophysiological mechanisms underlying hypogonadism involve either gonadal failure or failure of the hypothalamus or pituitary to release adequate stimulatory hormones for the gonadal sex hormone production [3].

Gonadal failure may be referred to as primary hypogonadism or hypergonadotropic hypogonadism, while insufficiency of hypothalamus and/or pituitary gonadotropin release results in hypogonadotropic hypogonadism, also known as secondary or central hypogonadism [4]. Secondary hypogonadism may occur as a result of conditions such as hemochromatosis, pituitary tumors, and exposure to drugs such as corticosteroids or opioids [5].

Altered functioning of the hypothalamic-pituitary-adrenocortical (HPA) axis may be indicative of the nature of the motivational modifications that accompany addiction and addiction vulnerability. The HPA axis is an important system to examine in relation to familial risk or existing addiction. Acute intake of alcohol and nicotine creates stress-like cortisol responses, and their continued use may dysregulate the HPA axis. Excessive intake of alcohol and nicotine may trigger changes in frontal-limbic interactions and may account for HPA response differences [6].

Drug abuse is commonly referred to as the nontherapeutic use of a drug product or substance for a desired psychological or physiological effect [7]. Drug addiction is increasingly becoming a major worldwide medical and social problem that is prevalent in both developed and developing countries [8].

Drug abuse has increased dramatically in the world today, especially in developing countries such as Ethiopia. Drug abuse is often associated with harmful effects and causes certain difficulties not only for individuals who abuse the drug but also for their parents, peers, and society as a whole. The effects of different drugs of abuse on the endocrine system are multiple and complex. The type, duration, and pattern of exposure; levels of intoxication and withdrawal; and coexisting medical problems often predict the degree of endocrine disruption [9, 10].

The WHO estimate for 2014 shows a global burden of 185 million drug abusers. It is estimated that one in 20 adults, or a quarter of a billion people aged 15 to 64 years, uses at least one drug in 2014. In addition, more than 29 million people who use drugs suffer from drug abuse disorders. The number of drug-related deaths worldwide has also remained stable with an estimated 207,400 deaths in 2014, corresponding to 43.5 deaths per million people aged 15–64. Khat is the most commonly used drug in Africa and particularly in the Horn of Africa [11, 12].

The traditional practice of chewing khat is being changed to a complex scheme leading to multidrug use. Drug abuse has had an impact on serious societal issues such as social maladaptation, decrease labor productivity, and job losses. In addition, drug abuse leads to the development of tolerance and demand for high doses to achieve the desired stimulating effect and has severe adverse implications [13, 14].

There are limited studies in Africa on the gonadal and cortisol hormone profiles of drug abusers. Therefore, this study aims to assess the effects of abuse of khat, heroin, and marijuana on luteinizing hormone (LH), estradiol (E2), follicle stimulating hormone (FSH), testosterone, and cortisol hormone among adult male drug abuse.

## 2. Methods

### 2.1. Objective

To compare the median difference of gonadal and cortisol hormone levels between each of drug abuse (khat, marijuana, and heroin) and control groups.

To determine the correlation of different gonadal hormones (E2, LH, FSH, and testosterone) and cortisol hormone between each of drug abuse (khat, marijuana, and heroin) and control groups.

### 2.2. Study Design and Study Area

A case-control study was conducted from March to May 2017 at the Zewditu Memorial Hospital, Addis Ababa, Ethiopia. A total of 148 eligible males who were drug abused (marijuana, heroin, and khat) were included in the case group. At the same time, 23 healthy men control groups were selected from healthy volunteer blood donors by controlling age group with the case groups.

### 2.3. Inclusion and Exclusion Criteria

An adult male with experience in drug abuse (khat, marijuana, and heroin) over the past six months and apparently healthy male with no self-reported history of drug use was selected as a case group and control group, respectively. Study participants with a condition that has a significant impact on the endocrine system such as sexually transmitted disease, daily alcohol consumption, cancer, diabetes, severe medical conditions requiring pharmacological therapy, and any psychiatric illness were excluded. In addition, female drug abusers were excluded in our study area due to a very limited number of cases.

### 2.4. Data Collection, Quality Assurance, and Laboratory Methods

Information on the study participants was obtained using a semi-structured questionnaire by the interview method. The questionnaire included socio-demographic characteristics such as age, sex, occupational status, marital status, educational status, and residence. The serum samples were collected by controlling the collection time, and serum gonadal and cortisol hormones were analyzed by the electrochemiluminescence (ECL) immunoassay analysis in a Cobas e411 analyzer. The result was interpreted by their standard reference ranges of the test method (LH:1.24–7.8 IU/L, FSH:2–15 IU/L, testosterone:2.7–10.7 ng/dL, estradiol:10–40 pg/mL, and cortisol:10–20 *μ*g/dL). Daily maintenance and inspection of the reagent bottle and expiry date were performed on the Cobas e411 analyzer. Two levels of quality control (QC) samples were performed to evaluate the functionality of the instrument and reagent, and the results of QC were evaluated using the Levey–Jennings chart. All phases of quality assurance during laboratory analysis were performed in the National Reference Laboratory for Clinical Chemistry at the Ethiopian Public Health Institute (EPHI). The EPHI laboratory is accredited by the Ethiopian National Accreditation Office (ENAO) to conduct tests in accordance with ISO 15189: 2012, Quality and Competence Medical Laboratory Requirements (accreditation no. M 0025) by well trained and experienced laboratory professionals, and standard operating procedures was strictly followed by respective parameters.

### 2.5. Data Analysis and Interpretation

Data from the automated analyzers (Cobas e 411) and interview were coded and entered into EPI info data version 7 and analyzed by using SPSS version 20. We performed a graphical normality assessment (histogram) and Kolmogorov–Smirnov and the Shapiro–Wilk normality test for our data. The existence of outliers and normal distribution was checked before the beginning of statistical analysis; therefore, nonparametric statistical tests were performed for all data not normally distributed. Statistical analyzes such as mean, median, and frequency were performed for socio-demographic variables and hormone parameters, shown in numbers and percentages using tables and graphs. A nonparametric Mann–Whitney test was performed to compare the difference in gonadal and cortisol hormone parameters between each of drug abuse (khat, marijuana, and heroin) and control groups. Similarly, the relationship between the variables of FSH, LH, E2, testosterone, and cortisol were evaluated by a nonparametric Spearman correlation. Fisher exact test was also done to assess the association between socio-demographic variables and testosterone hormone among drug abusers. *P* < 0.05 was considered statistically significant.

## 3. Result

### 3.1. Socio-Demographic Characteristics of Study Participants

The median age for drug abusers and control groups was 27, and there was no statistically significant age difference between drug abusers and control group. The majority of study participants was 44.6% heroin and 41.2% marijuana abused. Most of the participants were within the age group of 20–24 years. Of the total (148) drug abusers, 115 (77.7%) were unmarried. In terms of educational background, 66 (44.6%) and 40 (27%) of drug users have completed in the primary and secondary school, respectively. In addition, 88 (59.5%) attendants were hawkers, street vendors, and casual labors (Table 1).

### 3.2. Comparison of Hormonal Parameters between Case and Control Groups

**Figure 1:**
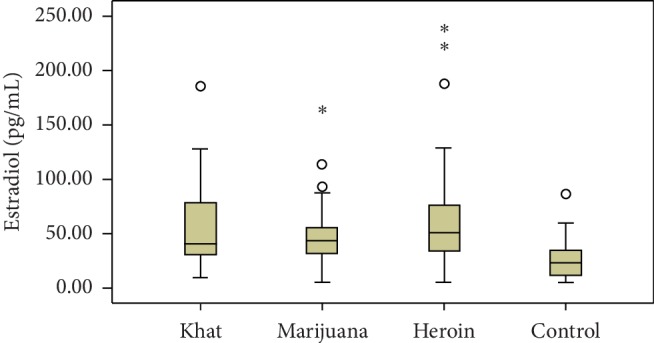
Box and whisker plot data comparison graph of median estradiol hormone levels among khat, marijuana, heroin, and control groups.

**Figure 2:**
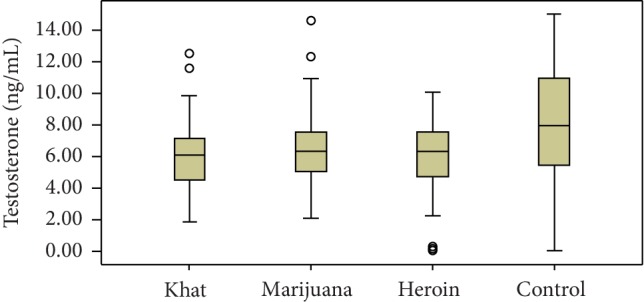
Box and whisker plot data comparison graph of median testosterone hormone levels among khat, marijuana, heroin, and control groups.

The effect of marijuana, heroin, and khat on the gonadal and cortisol hormone levels was evaluated in this study, and the values were compared with the control groups. Compared to control groups, serum testosterone levels were significantly decreased in khat (*P*=0.03), marijuana (*P*=0.011), and heroin (*P*=0.004) abusers. Similarly, the serum level of LH decreased significantly among each type of drug abuse compared with control groups. However, serum estradiol levels among khat, marijuana, and heroin users were increased significantly compared with control groups (Figures 1 and 2).

However, there was no statistically significant difference in cortisol and FSH hormone levels between each of drug abusers (khat, marijuana, and heroin) compared with the control group (Table 2).

### 3.3. Correlation of Hormone Parameters between Users of Heroin, Marijuana, and Chronic Khat and Control Group

There was a positive correlation between FSH (*P*=0.001) and LH (*P* ≤ 0.001) among users of heroin and marijuana. However, there is a lack of such a correlation among control groups and khat abuses. Furthermore, FSH was negatively correlated with estradiol in the abusers of heroin (*P*=0.033), marijuana (*P*=0.029), and khat (*P*=0.018). There was also a positive correlation between cortisol (*P*=0.005) and estradiol (*P*=0.012) hormone levels in the heroin and marijuana abusers. In addition, estradiol hormone level was positively correlated with testosterone (*P*=0.002) in the heroin abusers and negatively correlated with LH (*P*=0.039) in the khat abusers. However, none of the hormone parameters showed correlation in the control group (Table 3).

### 3.4. Testosterone and Associated Factors among Heroin, Marijuana, and Chronic Khat Users and Control Group

The study showed that there was no significant association between socio-demographic characteristics of drug abusers with the level of testosterone hormone (Table 4).

## 4. Discussion

The present finding showed significant reduction in LH (*P* = 0.021) and testosterone (*P* = 0.01) levels compared with the control groups among marijuana abusers [14]. This may be due to the temporary impairment of cannabinoids in the pituitary function reflected in decreased LH and FSH levels and hence reduced testosterone levels. The finding was consistent with a study conducted in Sudan that reported a significant decrease in LH and testosterone among marijuana abuses [15]. It was again supported by an animal model study conducted in India, which showed adverse effects on testes caused by intraperitoneal injection of cannabis extract at low doses. And, the histology finding revealed significant reduction in tubular diameter and detrimental changes in the seminiferous epithelium of the testes, resulting in lower serum testosterone and pituitary gonadotropin (LH and FSH) levels [16].

In the current study, the decrease in testosterone among marijuana users is in agreement with the Nigerian studies [17, 18]. However, this contradicts with a study done in the US [19] on chronic marijuana users with no significant difference in testosterone level. The decrease in testosterone could be attributed to inhibition of the gonadotrophin releasing hormone in the hypothalamus by 9-tetrahydrocannabinol (THC) [20]. These contradictory findings may be due to the difference in socio-demographic factors, the chronic use of marijuana, genetic differences, and the difference in the test method used.

However, the decrease in testosterone shown in khat users in our study contradicts the study done in Saudi Arabia [21], which resulted in an increase in testosterone in khat users. It was previously reported that cathinone, psychoactive alkaloid ingredients in khat, is responsible for the decrease in testosterone concentration [22]. The dose-dependent effects of khat on testosterone reported that low concentrations of khat extract increased significantly, while high concentrations suppressed the production of testosterone [23]. This may imply that the decline in testosterone found in our study may be due to high dose consumption of khat, and note that, in our study, 72.3% of study participants were high dose users.

The level of plasma cortisol was higher among marijuana abusers relative to the control group and was supported by the studies [24, 25]. In addition, the U.S. research showed high plasma cortisol concentrations in a dose-dependent way, but frequent users showed blunt increases relative to healthy controls [26]. These findings indicate that HPA axis activity may be dysregulated by heavy marijuana use. Marijuana modifies HPA axis function by affecting CRH release either directly through CB1-mediated effects in the paraventricular nucleus on CRH neurons or indirectly through other hypothalamic mechanisms [27].

However, the results of the present study showed that the level of serum cortisol was lower in khat users than in normal controls, and this is inconsistent with the study conducted in Saudi Arabia [21] and in animal studies [28, 29]. Its low concentrations may be partially explained by decreased cortisol-releasing hormone (CRH) synthesis and/or release at the hypothalamus. These findings indicate and extend previous findings by showing the impacts of cathinone on hypothalamus-hypophyses-adrenocortical axis and mesocorticolimbic systems. In addition, it may also be due to the decreased responsiveness of its receptors to the pituitary and adrenal gland due to exposure to cathinone [28]. A further hypothesis trying to explain the observed low serum cortisol levels is the potential involvement of the extrahypothalamic CRH systems involved in the behavioral excitement [30].

The abuse of khat results in a significant increase in E2 (*P* ≤ 0.03). This finding was inconsistent with an animal study showing that the khat abuse can cause increases in testosterone and E2 [31]. This may be due to the conversion of testosterone to estradiol by aromatase or may be due to testosterone receptors of estrogen saturates in the hypothalamus, which may shut down normal testosterone testicular production [32].

In our study, heroin abuses showed an increase in estradiol, whereas a decrease in LH and testosterone. This finding was in contrast with a study conducted in Pakistan and Iran [33, 34] that reported a nonsignificant difference between heroin addicts and control groups. The use of heroin is thought to inhibit the production of gonadotropin-releasing hormone, which reduces the release of luteinizing hormones and subsequently reduces the production of testosterone [35]. These different findings may be due to different Iranian crack (heroin) composition. Though it is opioid-based, it has a complex and different composition.

In our findings, the decreased serum concentrations of testosterone and LH among heroin users were consistent with studies [14, 36] that reported decreased serum testosterone concentrations in heroin abusers with no consistent abnormalities in other hormones. This decrease in testosterone among heroin users may be due to the effect of heroin on the hypothalamus and pituitary hormone secretions and the direct effects on pituitary gonadotropin-releasing cells via kappa and mu opioid receptors [14, 37].

## 5. Conclusion

Our study found that luteinizing hormone and testosterone were significantly lower in the drug abuser groups compared with control groups. However, there was no significant difference in FSH between drug abuse and control groups. Therefore, physicians should consider screening the gonadal hormones among drug abusers in addition to other metabolic and psychiatric support.

## Figures and Tables

**Table 1:** Socio-demographic characteristics of the study participants visited Zewditu Hospital, Ethiopia, in 2017.

Variables	Category	Frequency (*n*)	Percentage (%)
Age (years)	15–19	16	10.7
	20–24	44	29.85
	25–29	34	22.9
	30–34	25	16.9
	35–39	14	9.4
	40–44	7	4.7
	45–49	5	3.3
	>50	3	1.9

Marital status	Married	33	22.3
	Unmarried	115	77.7
	Total	148	100

Educational status	Primary	66	44.6
	High school	40	27
	Preparatory	23	15.5
	College and above	19	12.8
	Total	148	100

Occupational status	No job	28	18.9
	Waiter/bar manager/hotel	1	0.7
	Tourism/travel agent/tour guide	3	2
	Mechanic/factory worker/labor	5	3.4
	Health professional/teacher/banker	9	6.1
	Businessman	1	0.7
	Hawker/street vendor/casual labor	88	59.5
	Musician/dancer/performer	2	1.4
	Driver (private/taxi/truck)	7	4.7
	Drug dealer	2	1.4
	Watchman/security guard	2	1.4
	Total	148	100

**Table 2:** Gonadal and cortisol hormone parameters between drug abuse and control groups at Zewditu Hospital, Ethiopia, in 2017.

Type of drug abuse	*N* (%)	Hormonal parameters	Median (control group)	Median (drug abuser)	*P* value
Khat	21 (14.2)	Cortisol	15.02	16.23	0.934
		E2	22.59	39.42	0.003^*∗*^
		FSH	3.22	3.24	0.869
		LH	6.16	4.97	0.003^*∗*^
		Testosterone	7.95	6.07	0.03^*∗*^

Marijuana	61 (41.2)	Cortisol	15.02	14.01	0.120
		E2	22.59	43.93	<0.001
		FSH	3.22	3.01	0.055
		LH	6.16	5.01	0.021^*∗*^
		Testosterone	7.95	6.31	0.01^*∗*^

Heroin	66 (44.6)	Cortisol	15.02	17.2	0.120
		E2	22.59	40.15	<0.001
		FSH	3.22	2.37	0.055
		LH	6.16	5.65	0.021^*∗*^
		Testosterone	7.95	6.62	0.004^*∗*^

Cortisol (*μ*g/dL), E2 (pg/mL), FSH (lu/L), LH (lu/L), and testosterone (ng/dl) ^*∗*^Mann–Whitney test significant (*P*value <0.05). Reference range (LH: 1.24–7.8 IU/L, FSH: 2–15 IU/L, testosterone: 2.7–10.7 ng/dL, estradiol: 10–40 pg/ml, and cortisol: 10–20 *μ*g/dl).

**Table 3:** Correlation of hormone parameters in heroin, marijuana, and khat abuse and control group.

Hormone parameters	Heroin (rho)	Marijuana (rho)	Khat (rho)	Control (rho)
FSH vs LH	0.406^*∗*^	0.519^*∗*^	0.292	−0.274
FSH vs E2	−0.262^*∗*^	−0.280^*∗*^	−0.356^*∗*^	−0.356
Cortisol vs E2	0.343^*∗*^	0.320^*∗*^	0.236	−0.274
E2 vs testosterone	0.367^*∗*^	0.210	0.151	0.266
E2 vs. LH	−0.106	−0.119	−0.156^*∗*^	0.003

rho: Spearman correlation; ^*∗*^significant correlation (*P*value <0.05).

**Table 4:** Association of testosterone hormone levels with socio-demographic variables and the type and dose of drug abuse among drug abuses attended Zewditu Hospital.

Variables	Category	Testosterone		*P* value	d*f*	Fisher exact test
		Normal	Low			
Age (years)	15–19	15 (10.1)	1 (0.6)	0.378	7	7.396
	20–24	39 (26.4)	5 (3.4)			
	25–29	28 (18.9)	6 (4)			
	30–34	21 (14.2)	4 (2.7)			
	35–39	13 (8.8)	1 (0.6)			
	40–44	4 (2.7)	3 (2)			
	45–49	4 (2.7)	1 (0.6)			
	>50	2 (1.3)	1 (0.6)			

Marital status	Married	26 (17.5)	7 (4.7)	0.245	1	1.542
	Unmarried	100 (67.6)	15 (10)			

Educational status	Primary	56 (37.8)	10 (6.7)	0.385	4	3.288
	High school	35 (23.6)	5 (3.4)			
	Preparatory	17 (11.5)	6 (4)			
	College and above	18 (12.2)	1 (0.6)			

Occupational status	No job	24 (16.2)	4 (2.7)	0.068	10	14.08
	Waiter/bar manager/hotel	1 (0.6)	0 (0)			
	Tourism/travel agent/tour guide	2 (1.3)	1 (0.6)			
	Mechanic/factory worker/labor	5 (3.4)	0 (0)			
	Health professional/teacher/banker	8 (5.4)	1 (0.6)			
	Businessman	1 (0.6)	0 (0)			
	Hawker/street vendor/casual labor	77 (52)	11 (7.4)			
	Musician/dancer/performer	0 (0)	2 (1.3)			
	Driver (private/taxi/truck)	5 (3.4)	2 (1.3)			
	Drug dealer	2 (1.3)	0 (0)			
	Watchman/security guard	1 (0.6)	1 (0.6)			

Dosage of drug	High	94 (63.5)	15 (10)	0.285	2	2.913
	Low	32 (21.6)	7 (4.7)			

Type of drug abuse	Khat	18 (12.2)	3 (2)	0.906	2	0.256
	Marijuana	51 (34.5)	10 (6.7)			
	Heroin	57 (38.5)	9 (6)			

## Data Availability

The data used to support the findings of this study are included within the article.

## Abbreviations

ACTH: Adrenocorticotrophic hormone
EPHI: Ethiopian Public Health Institute
E2: Estradiol
FSH: Follicle stimulating hormone
HPA: Hypothalamic-pituitary-adrenocortical
HPG: Hypothalamic-pituitary-gonadal axis
LH: Luteinizing hormone
QC: Quality control
THC: Tetrahydrocannabinol
WHO: World Health Organization.
